# Composites of Reduced Graphene Oxide Based on Silver Nanoparticles and Their Effect on Breast Cancer Stem Cells

**DOI:** 10.3390/bioengineering12050508

**Published:** 2025-05-11

**Authors:** Babu Vimalanathan, Devasena Thiyagarajan, Ruby Nirmala Mary, Magesh Sachidanandam, Savarimuthu Ignacimuthu, Dhanavathy Gnanasampanthapandian, Johnson Rajasingh, Kanagaraj Palaniyandi

**Affiliations:** 1 Crystal Growth Centre, Anna University, Chennai 600025, Tamil Nadu, India; babu_antony12@yahoo.com; 2 Centre for Nanoscience and Nanotechnology, Anna University, Chennai 600025, Tamil Nadu, India; tdevasenabio@gmail.com; 3 Department of Biotechnology, Periyar Maniammai Institute of Science and Technology, Thanjavur 613403, Tamil Nadu, India; rubynano87@gmail.com; 4 Department of Virology, King Institute of Preventive Medicine and Research, Chennai 600032, Tamil Nadu, India; mageshking78@gmail.com; 5 Xavier Research Foundation, St Xavier’s College, Palayamkottai, Tirunelveli 627002, Tamil Nadu, India; imuthus@hotmail.com; 6 Cancer Science Laboratory, Department of Biotechnology, SRM Institute of Science and Technology, Chennai 603203, Tamil Nadu, India; dhanavag@srmist.edu.in; 7 Department of Bioscience Research & Medicine-Cardiology, The University of Tennessee Health Science Center, Memphis, TN 38163, USA

**Keywords:** nanocomposites, silver nanoparticles, graphene oxide, cancer stem cells, Raman spectroscopy, mammosphere

## Abstract

Graphene and its related nanocomposites have garnered significant interest due to their distinct physiochemical and biological properties. In this study, reduced graphene oxide–silver hybrid nanostructures were synthesized for applications in biomedical nanotechnology, particularly in targeting cancer stem cells (CSCs). A range of analytical techniques, such as X-ray diffraction (XRD), Raman spectroscopy, Fourier-transform infrared spectroscopy (FTIR), scanning electron microscopy (SEM), and UV–visible absorption spectroscopy (UV–VIS), were employed to characterize graphene oxide (GO), reduced graphene oxide (rGO)–silver nanoparticles (AgNPs), and their composite structures. The GO-rGO-AgNPs exhibited potent anticancer properties as evidenced by cell culture assays, spheroid formation assay, and quantitative RT-PCR analysis. Treatment of breast cancer cells (MCF-7) with GO, rGO, and AgNPs significantly reduced cell proliferation and mammosphere formation. Furthermore, these treatments downregulated the expression of marker genes associated with CSCs in MCF-7 cells. Among the tested materials, rGO-AgNP, sodium citrate-mediated GO-AgNP, and rGO-AgNP nanocomposites demonstrated superior inhibitory effects on cell survival compared to GO alone. These findings suggest that these nanocomposites hold promise as effective and non-toxic therapeutic agents for targeting cancer cells and CSCs, thereby offering a novel approach to cancer treatment.

## 1. Introduction

Graphene, a single layer of sp2-hybridized carbon atoms arranged in a two-dimensional honeycomb lattice, has drawn significant attention in scientific and industrial applications due to its exceptional properties [1,2,3,4]. These include remarkable mechanical strength, high electron mobility, superior electronic transport, and excellent electrical conductivity. As a result, graphene has found applications in nanocomposites [5,6], nanoelectronics [7,8], and energy storage devices [9,10]. In addition, graphene nanoparticles have recently been increasingly explored for biological applications [11,12], such as drug delivery [13,14], cellular imaging, antibacterial activity [15,16,17], biosensing [18,19], and anticancer therapies [20,21,22,23,24,25].

Graphene oxide (GO), a hydrophilic derivative of graphene, produces stable and homogeneous, colloidal suspensions in aqueous and polar solvents due to its oxygen-containing functional groups. It serves as a precursor for reduced GO (rGO) through chemical deoxygenation [7,8]. With its large surface area, abundant functional groups, and high water solubility, GO has demonstrated potential in numerous biomedical applications, including drug delivery, biosensing, antibacterial, antiplatelet activity, anticancer therapies, and scaffolding for tissue engineering [26,27]. Notably, graphene materials can enhance cell adhesion, stimulate cell proliferation, and promote differentiation, serving as effective scaffolds in regenerative medicine [26,28].

Graphene substrates have been shown to promote neurite outgrowth in mouse hippocampal cells, whereas rGO sheets induce neurite genesis of PC12 cells [28,29]. GO suspensions further promotes the differentiation of mice embryonic stem (ES) cells and the osteogenic differentiation of human mesenchymal stem cells (MSCs) [30,31]. Beyond graphene, silver nanoparticles (AgNPs) have also been extensively studied due to their unique properties, including high surface areas and nanoscale dimensions. These characteristics make AgNPs useful in energy science, optics, electronics, catalysis, nanobiotechnology, and nanomedicine, especially as antibacterial agents [32,33]. Several studies have shown that AgNPs act as anti-angiogenic and anticancer agents in retinal endothelial cells [34]. AgNP treatment had significant effects on cytotoxicity observed in human lung fibroblasts (IMR-90), human glioblastoma cells (U251), and endothelial cells [35,36].

The combination of GO with AgNPs has gained significant interest because of the complementary and enhanced properties of these materials [37,38,39]. GO sheets provide a stable platform for the controlled binding and release of AgNPs, resulting in improved antibacterial and anticancer activities [40,41,42]. This synergy is particularly relevant for developing innovative therapeutic agents targeting multiple factors that contributed in cancer progression, metastasis, and resistance to conventional treatments [43,44].

Cancer therapy remains a significant challenge due to multidrug resistance (MDR) and the role of cancer stem cells (CSCs) in tumor recurrence and metastasis [45]. Nanoparticle-mediated drug delivery offers a promising approach to address these issues by precisely targeting cancer cells and minimizing side effects [46]. In this study, we synthesized and characterized GO and rGO–silver nanocomposites using sodium citrate and evaluated their potential cytotoxic effects on breast CSCs. This research aims to contribute to the development of novel therapeutic agents for overcoming the limitations of current cancer treatments.

## 2. Materials and Methods

### 2.1. Chemicals

Graphite powder, laboratory grade Thiourea, NaOH, KMnO_4_, anhydrous ethanol, 98% H_2_SO_4_, 36% HCl, and 30% hydrogen peroxide (H_2_O_2_) aqueous solution were procured from Sigma Aldrich, Mumbai; they were employed with no additional refinement. Trypsin, water-soluble tetrazolium salt (WST-8), dimethyl sulfoxide (DMSO), and DMEM were bought from Hi Media Laboratories. Fetal bovine serum (FBS) was acquired from Cistron Laboratories. Deionized water was used to prepare aqueous solutions. All reagents and chemicals were purchased from Sigma-Aldrich (St Louis, MO, USA). The breast cancer cell line MCF-7 was purchased from NCCS (Pune, India). DMEM cell culture media, trypsin-EDTA, and antibiotics were procured from HiMedia (Bangalore, India). MTT and DMSO were procured from Sigma-Aldrich (USA). Fetal bovine serum was purchased from Thermoscientific, Waltham, MA, (USA). Ultra-low attachment plate and Mammocult media were purchased from Stem Cell Technologies, Vancouver, BC, (Canada). The total RNA isolation (RNAeasy mini) kit and the DNAase kit were obtained from Qiagen, Hilden (Germany). The SYBR master mix was procured from Promega, Madison, WI (USA).

### 2.2. Preparation of Silver Nanoparticles

AgNP synthesis was performed according to a procedure that has been documented previously [47]. In brief, AgNPs were synthesized by soaking 7.5 mM sodium citrate (1.10 g) in 500 mL water (Sigma-Aldrich) containing 5 mM AgNO_3_ (0.424 g) for 12 h at 60 °C. The production of AgNPs in the reaction mixture was responsible for the color shift from colorless to black. The reduction of the silver ions was monitored spectrophotometrically at 420 nm. Further characterization of the synthesized silver nanoparticles were performed as described previously [48].

### 2.3. Preparation of Graphene Oxide (GO) Sheets

Graphene oxide was produced by the modified Hummers method [49]. Graphite powder (1 g) was mixed in 90 mL of 98% concentrated sulfuric acid. The mixture was stirred in an ice bath for 30 min (10 °C). A further 6 g of KMNO_4_ was added and stirred in an ice bath for 18–24 h until a murky brown color paste was obtained (10 °C), Later, 500 mL deionized water was added and stirred for 1 h at 10 °C. Following this, 5 ml of 30% H_2_O_2_ was added, mixed, and stirred well using a magnetic stirrer overnight in an ice bath. The hue shifted from murky brown to dark brown. Then, 200 mL of diluted HCl (HCl 1:10 H_2_O) was added, mixed well for 30 min using a magnetic stirrer. The color became yellowish brown. This was centrifuged at room temperature at 5000 rpm for 5 min. The pellet was allowed to settle down. The pellet was washed more than 5 times with de-ionized water to remove any residual ions. The graphene oxide pellet was suspended in 50 mL of de-ionized water and sonicated for 30 min. The sonicated graphene oxide nanosheets solution was dried at 60 °C for 24 h in vacuum oven.

### 2.4. Preparation of rGO via Thermal Reduction

The GO was heated in an N_2_ environment (flow rate of 0.12 m^3^/h) in an oven at 750 °C and then at 950 °C to achieve thermal reduction. The GO was maintained at this temperature for 45 s [50,51] after reaching the target temperature.

### 2.5. Preparation of GO-Ag Nanocomposite

The silver-containing solution, which included AgNO_3_ (5 mM, 200 mL) and sodium citrate (7.5 mM, 200 mL), was mixed with 0.5 g of GO powder. After the mixture was finalized, the solution was treated with pulsed microwave-assisted (MA) synthesis. To ensure the development of silver seeds deposited on the GO surface, the solution was placed in a microwave oven (Tatung Co., Taipei City, Taiwan; 900 W, 2.45 GHz) at 160 °C for 5 min. After preparation, the GO-Ag solution was dried overnight at 60 °C in a vacuum oven [52].

### 2.6. Preparation of rGO-Ag Nanocomposite

The silver-containing solution, which contained AgNO_3_ (5 mM, 200 mL) and sodium citrate (7.5 mM, 200 mL), was mixed with 0.5 g of rGO powder. After the mixture was finalized, the solution was treated with pulse MA synthesis. To ensure the development of silver seeds deposited on the GO surface, the solution was placed in a microwave oven (Tatung Co., Taipei City, Taiwan; 900 W, 2.45 GHz) at 160 °C for 5 min. After preparation, the rGO-Ag solution was dried all night at 60 °C in a vacuum oven [42].

### 2.7. Characterization of Nanocomposites

The previously mentioned techniques were used to characterize GO, rGO, and the rGO–Ag nanocomposites [53]. The ultraviolet–visible (UV–vis) spectra GO, rGO, and rGO–Ag were recorded by using an OPTIZEN POP-V spectrophotometer (Mecasys Co., Seoul, Republic of Korea). XRD measurements were performed with a Rigaku Ultima IV using CuKα radiation at a wavelength of 1.54 Å and a 10 mm Cu target slit. Raman spectral measurements were performed by using a Renishaw PLC Raman spectrometer at an excitation wavelength of 514 nm (argon ion laser source).

The materials were dried and powdered with KBr pellets and examined with a Bruker Tensor 37 for FTIR analysis. Random micrographs were taken with an accelerating voltage of 15 kV at different points on a clean glass slide to which a droplet of the nanoparticle solution was transferred for SEM (Carl Zeiss, Overkochen, Germany).

### 2.8. Cell Viability Assay

MCF-7 cells were cultured in DMEM supplemented with 10% fetal bovine serum and 1% penicillin and streptomycin in an atmosphere of 5% CO_2_ at 37 °C. The cell viability was assessed by using trypan blue exclusion method. MCF-7 cells (1 × 10^4^) were plated in 96-well plates and incubated overnight for attachment. MCF-7 cells were treated with different concentrations (1, 10, 50, and 100 µM) of nanoparticles (GO, rGO, AgNPs) and their combinations for 48 h. After 48 h, the nanoparticles were carefully removed, the MTT reagent was added and incubated for 3 h at 37 °C in a CO_2_ incubator. The MTT media were discarded, and the formazon crystals were dissolved in dimethyl sulfoxide. The plates were read at 570 nm in an ELISA plate reader (BioTek, Winooski, Vermont, USA).

### 2.9. Mammosphere Formation Assay

The mammospheroid assay technique was used as reported previously. MCF-7 cells were transferred to single cell suspensions with trypsin. The cells (500 cells/well) were mixed in mammocult media and plated in an ultra-low attachment plate (12 wells). The MCF-7 cells were incubated overnight. Subsequently, the cells were treated with nanoparticles (GO, rGO, AgNPs) at a dose of 10 µM and allowed to form mammospheres for 7 days. The mammospheres were imaged in an inverted phase contrast microscope (EVOS M5000) and the number of spheroids was counted.

### 2.10. Quantitative RT-PCR

The MCF-7 cells were plated in 10 cm^2^ Petri dish and allowed to grow to approximately 70% confluence. MCF-7 cells were treated with the nanoparticles (GO, rGO, AgNPs) at 10 µM for 24 h. After treatment, the cells were trypsinized to obtain a cell pellet. The cell pellet was used to isolate total RNA (RNeasy mini kit, Qiagen, Germany). The total RNA was treated with the DNase kit from Invitrogen, MD, (USA). The total RNA (2 µg) was converted to cDNA using the cDNA synthesis kit according to the manufacturer’s instructions (iScript, BioRad, CA, USA). The template DNA was used at 95 °C for hot-start, 62 °C for annealing, and 60 °C for extension (QauntStudio 5, Thermoscientific, USA). A total of 35 cycles were repeated to amplify target genes, such as *CD24*, *CD44*, *ALDH1*, *SOX-2*, *NANOG*, *OCT-4*, *EPCAM*, *LGR5*, and GAPDH. The ΔΔCT values were converted into differences between the nanoparticle-treated groups.

### 2.11. Statistical Analysis

All three independent experimental data were analyzed statistically by one way analysis of variance (ANOVA) by using IBM-SPSS.30 statistical software. *p* < 0.05 was considered to be statistically significant when compared with the control groups. In between groups were also compared using Tukey HSD and Duncan test.

## 3. Results

### 3.1. UV–Vis Spectroscopy

**Figure 1:**
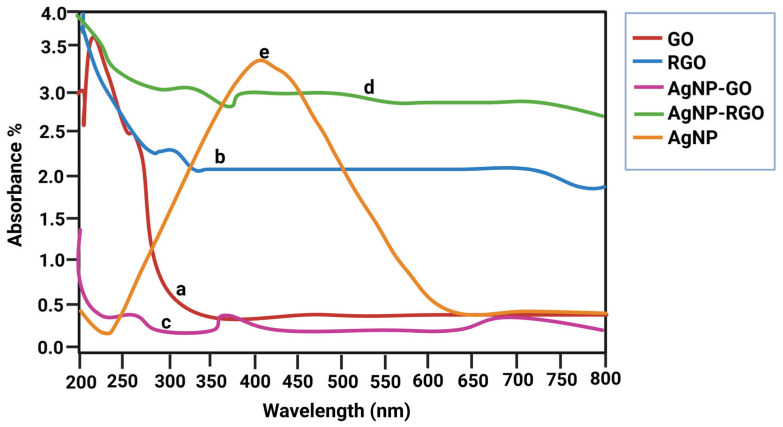
–UV–vis spectra of (a) GO, (b) rGO, (c)GO-AgNP, (d) rGO-AgNP, (e) AgNP3.

GO, rGO, and AgNO_3_ were combined to prepare the nanocomposite, and sodium citrate was used as a stabilizing and reducing agent. The UV–vis spectra of GO, rGO, and the GO-Ag rGO-Ag nanocomposites are shown in shown in Figure 1 a–e. At 230 and 292 nm, GO showed two distinct peaks representing the π–π* transitions of aromatic C–C bonds and the n–π* transitions of C = O bonds. The UV–visible spectra also confirmed the presence of GO-AgNPs. As shown in Figure 1, GO exhibited a typical peak at 230 nm corresponding to the aromatic C = C bond, whereas AgNPs associated with the graphene layer showed a typical characteristic peak at around 410 nm, consistent with the AgNP formulation and surface plasmon resonance phenomena. The evolution of the rGO–Ag nanocomposite and the concomitant decrease in rGO and AgNO_3_ were demonstrated clearly by the disappearance of the characteristic peaks of rGO and the presence of a new band originating from AgNPs.

### 3.2. Powder X-Ray Diffraction (PXRD) Analysis

**Figure 2:**
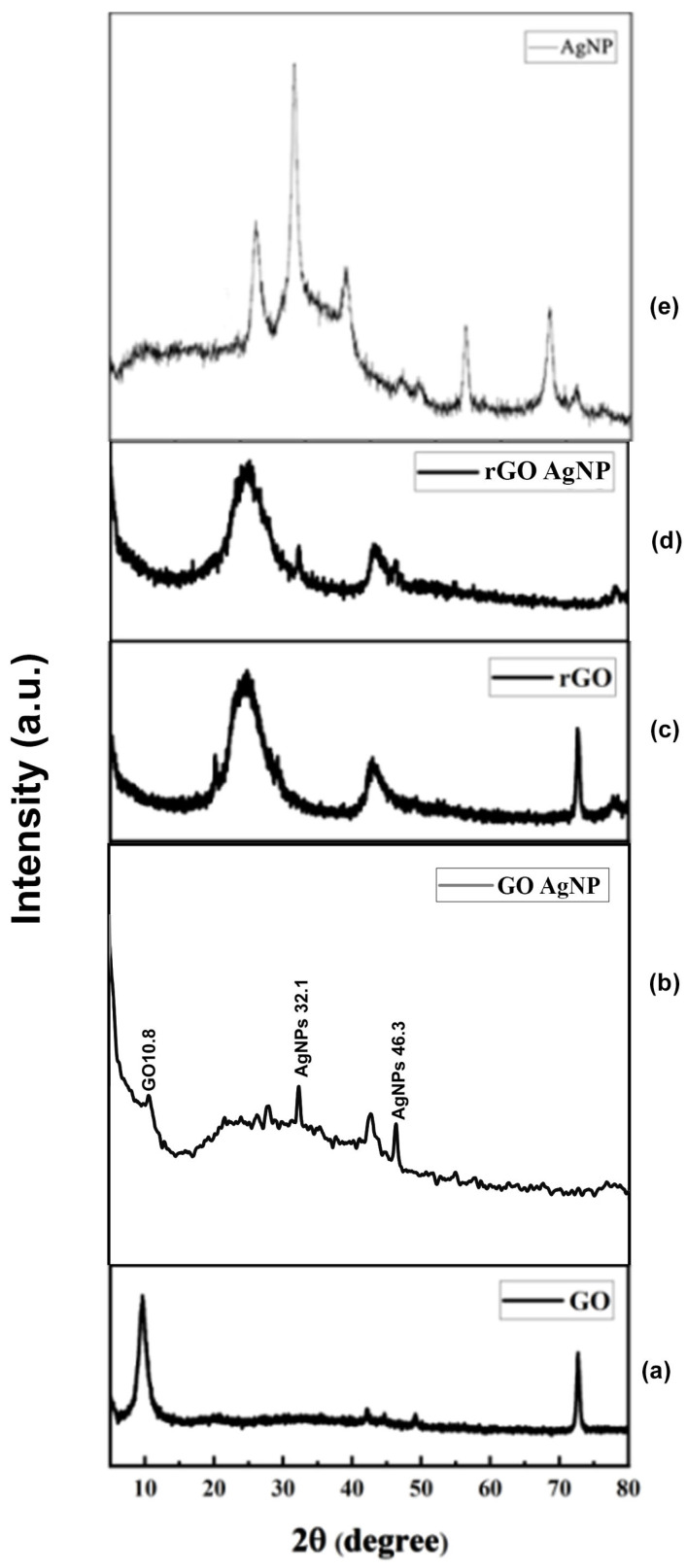
Powder XRD pattern of (**a**) GO, (**b**) GO-AgNP, (**c**) rGO, (**d**) rGO-AgNP, (**e**) AgNP composites.

XRD was used to further demonstrate that GO and GO-AgNP are crystalline materials. The PXRD pattern of artificially generated GO, AgNPs, and GO-Ag nanocomposites is shown in Figure 2. The plane (001) was represented by a strong 2θ = 10.19° value in the GO PXRD pattern. In addition, the (002) plane was reflected by a minor peak at 2θ = 20.93°. As oxygen-containing functional groups were added to graphite during its oxidation, the interlayer gap in GO increased compared to graphite.

However, as shown in Figure 2, a small variation in peak position towards a lower 2θ value confirms the incorporation of AgNPs in GO sheets. From Figure 2a,b, the peak at GO-10.9, GO-AgNP-10.8, 32.1 matches to the Figure 2e AgNP peak of 32.1 for comparison. Figure 2c,d shows that the peak at rGO-25.4, RGO-AgNP-25.4, 32.1 matches to the Figure 2e AgNP of 32.1 for comparison. However, in the rGO–Ag nanocomposites, in addition to the typical reflections of rGO (2θ = 25.4°), two unique reflections appeared at 40.1° and 45.3° in the diffractogram, corresponding to the (111) and (200) planes, respectively. This shows that metallic AgNPs were formed during the reduction.

### 3.3. SEM Analysis

**Figure 3:**
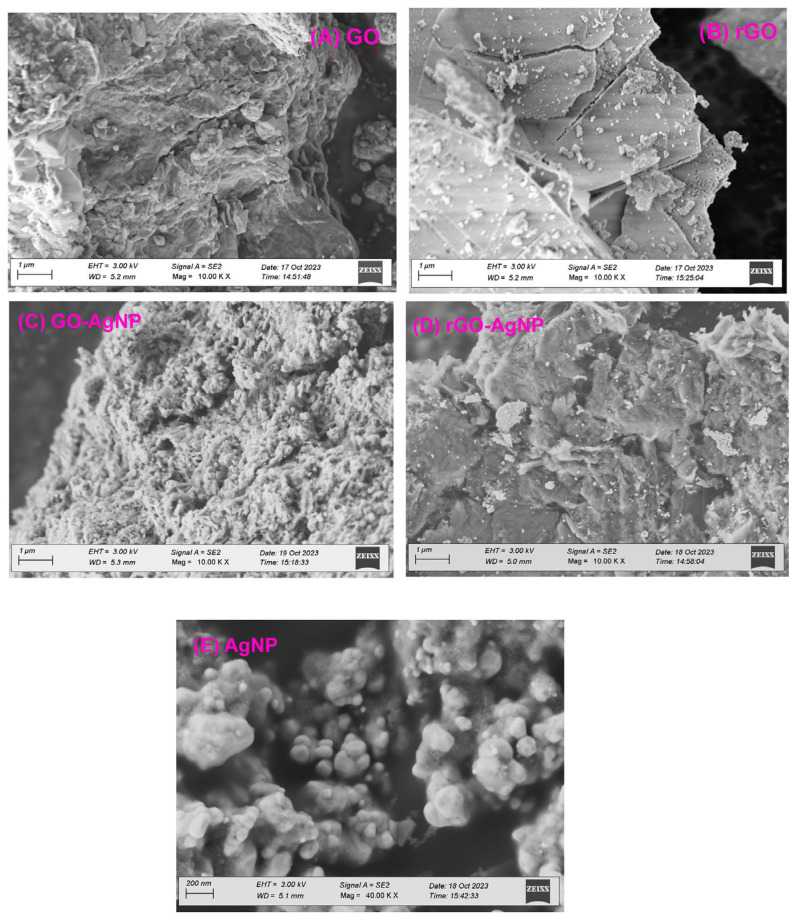
FE–SEM images of (**A**) GO, (**B**) rGO, (**C**) GO-AgNP, (**D**) rGO-AgNP, (**E**) AgNP with different magnifications.

The surface morphology of GO and the AgNP deposits on it were verified by using SEM. Stacking of the exfoliated nanosheets resulted in a well-packed, folded (and silky wave-like) morphology of the GO sheets. The surface morphology shows the appearance of a tightly folded curtain, with GO flakes overlapping instead of clumping. A remarkable morphological change was observed between GO and GO-AgNPs (Figure 3A–E). The AgNPs were uniformly dispersed on the surface of the composite nanosheets in distinct spherical shapes. The AgNPs appeared to be uniformly distributed over the GO sheets, as shown by the AgNPs distribution. Our results show that the graphite exfoliated significantly during the oxidation process and that sodium citrate effectively reduced GO and AgNO_3_ to GO-AgNPs (Figure 3).

In contrast to GO, rGO showed flaky, scale-like layers or transparent, rippling, silk-like waves. Typical SEM images of the prepared rGO-Ag hybrids are shown in Figure 3. Well-dispersed AgNPs were deposited on the graphene. Curled and wavy morphology was exhibited when Ag crystallites were generated on graphene surfaces as spacers between neighboring sheets in AgNPs-doped rGO. As spacers keeping adjacent sheets at a distance, the AgNPs were randomly distributed on the graphene sheets at a considerable distance from one another.

### 3.4. Fourier-Transform Infrared Spectroscopy (FTIR) Analysis

**Figure 4:**
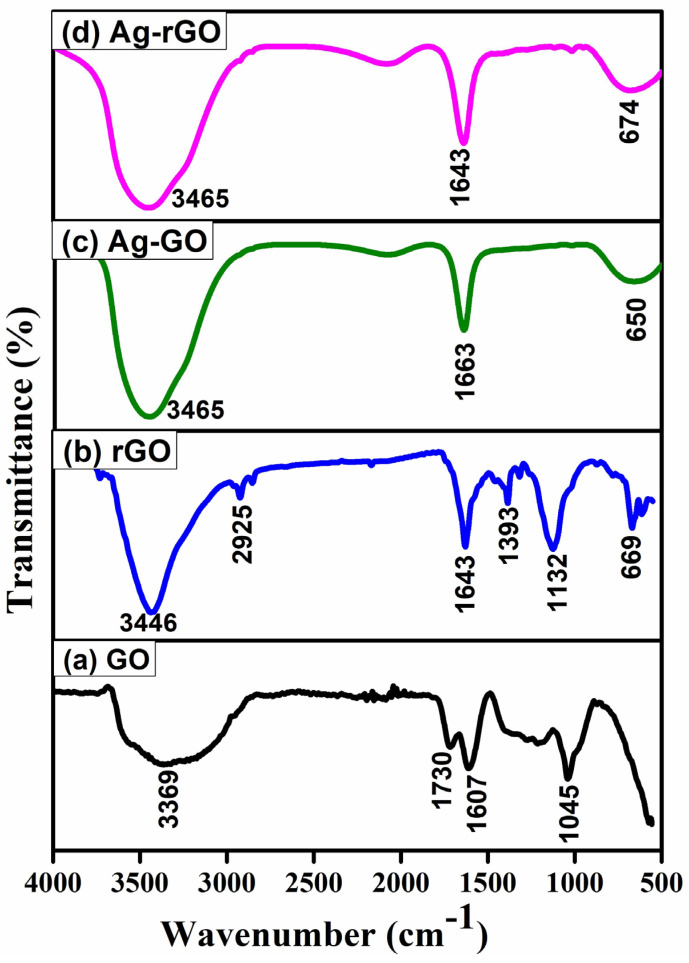
FTIR spectra of (**a**) GO, (**b**) rGO, (**c**) AgNP-GO, (**d**) AgNP -rGO- composites in the wave number ranging from 500 to 4000 cm^−1^.

FTIR analysis reveals the spectra of the synthesized GO and GO-Ag nanocomposite (Figure 4), which range between 4000 and 500 cm^−1^. An adsorption band corresponding to intermolecular H-bonding in GO was observed at 3437 cm^−1^, but this band disappeared in the GO-Ag nanocomposite. In addition, bands corresponding to C–H, C–O, and C–O were observed in 2072, 1636, and 1011 cm^−1^, respectively. After doping, the intensity of the C–O and ^−^OH bands decreased, indicating that AgNPs were anchored onto the GO surface. After doping, a blue shift was observed for these peaks.

### 3.5. Raman Spectroscopy

**Figure 5:**
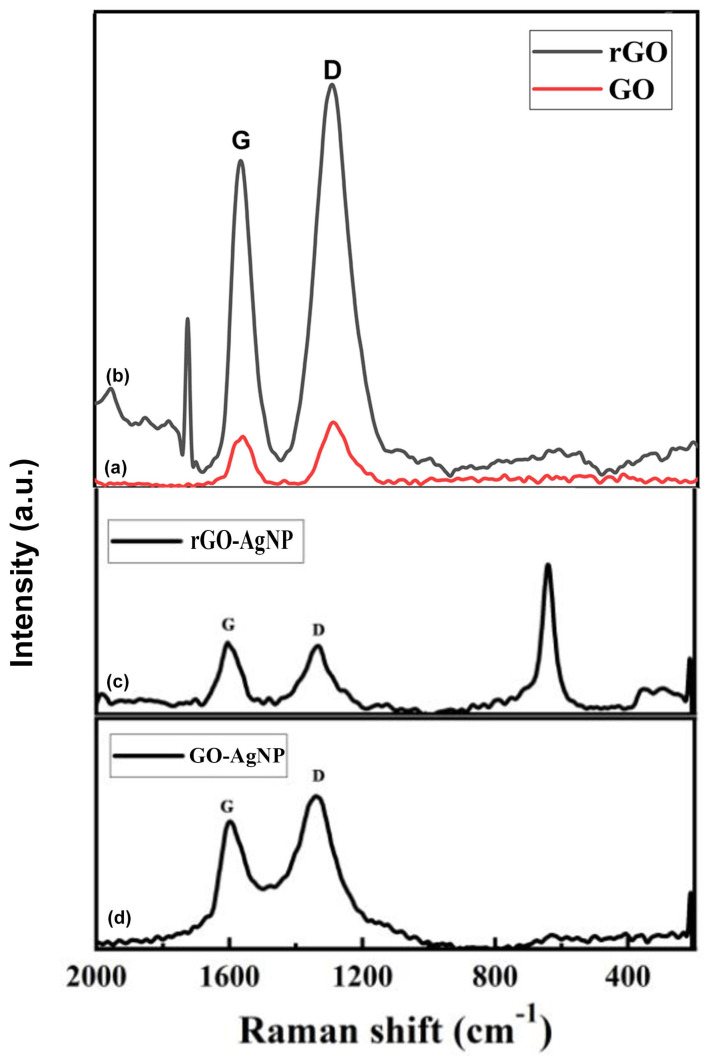
Raman spectra of (a) GO, (b) rGO, (c) GO-AgNP, (d) rGO-AgNP in the wave number ranging from 500 to 5000 cm^−1.^ Upper case G and D are bands.

The Raman spectra of the GO and GO-Ag nanocomposite are shown in Figure 5. The GO shows D and G bands at 1345 and 1575 cm^−1^, respectively, whereas the GO-Ag nanocomposite shows D and G bands at 1350 and 1590 cm^−1^, respectively.

The attachment of hydroxyl and epoxide groups to the basal plane of carbon resulted in structural defects, which were the source of the strong D peak. The accompanying Raman spectra show that the increased disorder of the rGO and rGO-Ag nanocomposite led to an enhancement of the D bands, while the enhanced isolated double bonds caused a broadening of the G bands of rGO and rGO-Ag. The G band of the rGO-Ag nanocomposite, which can be seen in Figure 5 at 1600 cm^−1^, was obviously shifted upward by 24 cm^−1^ compared to that of rGO (1584 cm^−1^), which is consistent with previous studies that show that the introduction of Au resulted in a shift of the G band through electron–phonon interaction.

### 3.6. Cell Viability Assay

**Figure 6:**
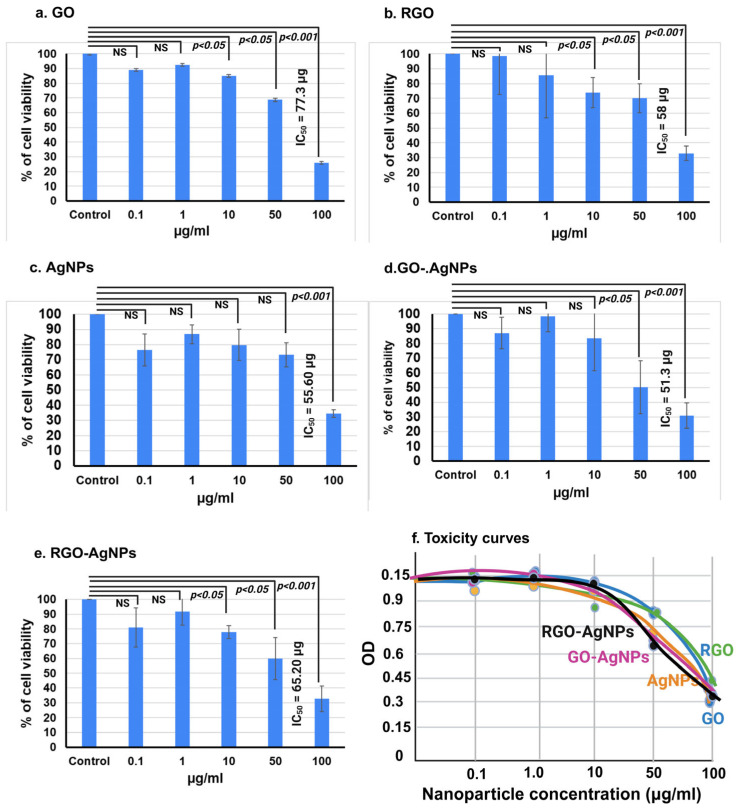
Nanomaterials inhibit the proliferation of MCF-7 breast cancer cells. MCF-7 were seeded in 96-well plates and treated with (**a**) GO, (**b**) rGO, (**c**) AgNP, (**d**) GO-AgNPs, and (**e**) rGO-AgNP, and (**f**) toxicity curves achieved using Hill coefficient, composites at different concentrations for 48 h. Cell viability was determined by using the MTT assay. The OD values of each treated group were compared with those of the control at the same time point. Results are shown as mean value ± SD of three independent experiments. *p* < 0.05 is considered to be statistically significant. NS means no significant.

Treatment of MCF-7 cells with GO nanoparticles at different concentrations (0.1, 1, 10, and 50 µM) significantly decreased the viability with increasing concentration from 1 µM and the lowest viability of 68.7% was observed in 50 µM GO. This clearly shows that GO nanoparticles induce cytotoxicity in a dose-dependent manner (Figure 6a–e). GO-AgNP treatment resulted in a 58.67% reduction in cell viability at 50 µM (Figure 6d). The antioxidant activity of the nanoparticles may play a role in cancer cell lines. Treatment with rGO nanoparticles alone reduced cell viability by 70.1% at 50 µM (Figure 6b). Treatment with AgNPs alone reduced cell viability by 82% at 50 µM in MCF-7 cells (Figure 6c). However, the combination of rGO-AgNP treatment resulted in 65% cytotoxicity at 50 µM (Figure 6e).

When MCF-7 cells were treated with AgNPs, the viability at 10 and 50 µM was more or less the same at 84% and 82.6%, respectively. This indicates that AgNPs have a weak anticancer effect compared to GO and rGO-AgNPs (Figure 6a–c).

### 3.7. Mammosphere Formation 

**Figure 7:**
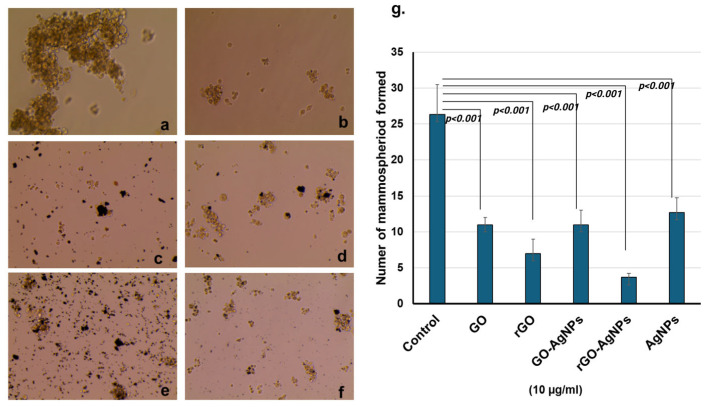
MCF-7-derived CSCs were allowed to form mammospheres for 7 days. (**a**) Control, (**b**) graphene oxide, (**c**) reduced graphene oxide, (**d**) GO-AgNP, (**e**) rGO-AgNP, (**f**) AgNPs. Treatment with nanomaterials reduced the size and number of mammospheres. Mammospheres treated with nanomaterials resulted in a lower number compared to the control, (**g**) mammosphere were quantified using EVOS M5000 microscopic software. The mammosphere formation was significantly reduced all the nanoparticle treatments. Mammosphere numbers were more significantly reduced in rGO and rGO-AgNPs treatment. *p* < 0.001 was considered to be more statistically significant.

Treatment of MCF-7 cells with the nanoparticles GO, rGO, GO-AgNPs, and rGO-AgNPs (10 µM) significantly reduced the mammosphere formation (Figure 7a–f). The mammosphere formation was more significantly reduced in rGO and rGO-AgNPs-treated cells when compared with GO and GO-AgNPs-treated cells (Figure 7g). In addition, the spheroid size is also significantly reduced by the treatment of nanoparticles.

### 3.8. Quantitative RT-PCR Analysis

**Figure 8:**
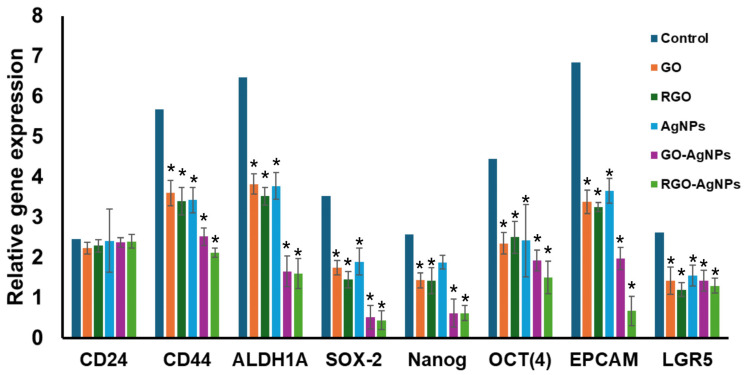
Treatment of MCF-7 cells with nanoparticles, significant reduction in stem cell marker gene expressions. ‘*’ Indicates statistically significant (*p* < 0.05) reduction in gene expression when compared with control gene expression.

Nanoparticle treatment reduced the expression of stem cell marker genes, such as *CD44*, *ALDH1A*, *SOX-2*, *NANOG*, *OCT4*, *EPCAM*, and *LAGR5* (Figure 8). The GO-AgNPs and rGO-AgNPs have the most significant inhibition on the gene expression of stem cell markers when compared with other nanoparticles. However, AgNP treatment alone did not affect gene expression of Nanog.

## 4. Discussion

The synthesized nanoparticles GO, rGO, and AgNPs were thoroughly characterized. The UV–vis spectra of rGO displayed a prominent absorption band at 261 nm, reflecting the significant restoration of the conjugated sp2 carbon network. Upon deposition of AgNPs on the rGO surface, a new peak appeared at 420 nm, which corresponds to surface plasmons resonance and confirms the successful incorporation of AgNPs on to the rGO surface [54,55,56,57,58]. AgNPs showed a prominent diffraction peak corresponding to the (111) plane at a 2θ value of 32.152°, consistent with the prior literature [59,60]. The GO-Ag nanocomposite displayed an interlayer spacing of 1.456 nm and peaks associated with both GO and AgNPs. The XRD pattern of the rGO–Ag nanocomposite revealed clear peaks corresponding to the (111) and (200) diffraction planes of face-centered cubic (fcc) Ag, supporting the crystalline nature of AgNPs [55,61,62,63]. These results align with earlier reports describing the structural configuration of GO/AgNPs composites synthesized through the reduction of AgNO3 and GO in the presence of reducing agents [9,20,24,55,64,65]. FTIR analysis provided further confirmation of interactions within the composites. The C–N stretching vibration observed in the TAPE-reduced rGO–Ag nanocomposite produced a distinct band at 1420 cm^−1^. Peaks at 657, 1011, 1636, 2070, and 3437 cm^−1^ also appeared in the FTIR spectra of the rGO-Ag nanocomposite, with intensities comparatively lower than those of GO, indicating the reduction process and interactions between residual hydroxyl groups and AgNPs [52,55,65,66].

Raman spectroscopy reveals that the G band, attributed to the E2g phonon of sp2 carbon atoms, and the D band, related to the breathing mode of k-point phonons with A1g symmetry, shifted upon AgNP deposition. For rGO, the G and D bands appeared at 1580 cm^−1^ and 1353 cm^−1^, respectively, suggesting structural changes and increased disorder due to AgNP intercalation [67,68,69]. The G band introduced the E2g phonon of the sp2 atoms of carbon, whereas the D band was attributed to the breathing mode of the k-point phonons with A1g symmetry.

The biological assessment shows that the GO-AgNPs composite demonstrated the most effective anticancer activity, with the lowest cell viability of 58.67% at a concentration of 50 µM compared to GO and AgNPs alone. The enhanced antioxidant and inhibitory effects of GO-AgNPs likely disturb cellular transcriptional and translational activity, though further experimental studies are required to elucidate the exact mechanisms [70,71]. In addition, GO, GO-AgNPs, and rGO-AgNPs effectively inhibited MCF-7 cancer cell proliferation and mammosphere formation, which is consistent with previous studies [72,73]. Interestingly, treatment with rGO and rGO-AgNPs significantly impacted the expression of cancer stem cell-associated gene expressions in breast and other types of cancers cell lines [74]. AgNPs have been known to induce cytotoxicity and inhibit stem cell proliferation at the transcriptional levels [75], while GO has been reported to promote the differentiation of hematopoietic stem cells [31] and myogenic progenitor cells [76].

## 5. Conclusions

The synthesized and characterized nanoparticles exhibit significant potential in targeting breast cancer cells and cancer stem cells. Specifically, rGO, GO-AgNPs, and rGO-AgNPs demonstrated promising anticancer effects by inhibiting MCF-7 cell proliferation and mammosphere formation. These findings suggest that nanocomposites play a critical role in reducing cancer stem cell self-renewal. Further in-depth studies, including toxicity assessments and targeted drug delivery strategies, are necessary to confirm their efficacy in preclinical models of breast cancer.

## Data Availability

The data available upon request. The original results stored in the office computer.
